# Olive orchard intensification compromises soil water erosion control in a semi-arid environment

**DOI:** 10.1371/journal.pone.0346675

**Published:** 2026-04-30

**Authors:** Luciano Gristina, Ettore Barone, Riccardo Scalenghe

**Affiliations:** 1 SAAF, University of Palermo, Palermo, Italy; 2 SEAS, University of Palermo, Palermo, Italy; University of Ferrara, ITALY

## Abstract

Olive cultivation is a key component of Mediterranean agriculture. Super-high-density (SHD) hedgerow orchards are expanding because they enable mechanization and early yields, yet their environmental impacts on semi-arid slopes remain insufficiently studied. We quantified soil erosion in a six-year-old SHD olive orchard in southwestern Sicily (15.6% slope) using δ^13^C depth profiles and erosion-pole height measurements, and we estimated the RUSLE cover-management factor (C) from field data and Sentinel-2 NDVI. The SHD orchard exhibited mean erosion of 29.4 Mg ha ⁻ ¹ yr ⁻ ¹, comparable to vineyards and markedly higher than rates reported for traditional olive systems. Canopy architecture provided ~38% ground cover, leaving ~62% bare soil, and frequent downslope tillage likely amplified rill formation and sediment redistribution. Independent methods converged on similar C_factors_ (0.38–0.39), indicating limited erosion control relative to traditional orchards. These site-specific results suggest that SHD orchards established on semi-arid slopes can substantially increase soil erosion risk. Conservation practices such as permanent cover crops, mulching/pruning-residue retention, and contour-aligned management could reduce erosion while maintaining productivity..

## Introduction

The cultivated olive tree (*Olea europaea* L. subsp. europaea var. europaea) is a leading oilseed crop in temperate areas and among the first domesticated fruit trees [1–2]. Beyond oil and table olives, olive agroecosystems provide multiple ecosystem services, including landscape value and cultural heritage, carbon sequestration, and hydrogeological protection [3–6]. Olive cultivation’s long history, combined with traits such as drought resistance, longevity, multifunctionality, and soil adaptability, has resulted in diverse planting systems across the Mediterranean Basin [7], with wide variation in tree density. The Mediterranean region accounts for approximately 98% of global olive cultivation [8]. Traditional systems range from 17–50 trees ha ⁻ ¹ (e.g., Tunisia) to 150–200 trees ha ⁻ ¹, while irrigated intensive systems reach 300–1000 trees ha ⁻ ¹ [9–10].

Driven by the effort to increase profitability and reduce production costs through earlier and higher yields, olive production has recently intensified toward dense, mechanically harvested hedgerow systems [11–12]. The most intensive form involves irrigated, fully mechanized hedgerow orchards exceeding 1,500 trees ha ⁻ ¹, termed “super-intensive” or super-high-density (SHD) systems [12]. Globally, SHD orchards have expanded from ~100,000 ha in 2011 [13] to >500,000 ha in recent estimates [14]; in Sicily, SHD is estimated to cover ~500 ha. Despite their rapid expansion, the sustainability implications of SHD systems remain insufficiently assessed, particularly regarding soil water erosion on sloping terrain. SHD design and management can increase erosion risk through downslope row orientation required for straddle harvesting, frequent tillage for weed control and evaporation reduction, and limited canopy-derived soil protection due to narrow, low hedgerows [5,15,16]. These features may lead SHD orchards to exhibit erosion risk levels comparable to vineyards on slopes, where row orientation and machinery traffic are major drivers [17–19].

This study aimed to: (i) quantify soil erosion in an SHD olive orchard using δ^13^C depth profiles and erosion-pole height measurements; (ii) estimate the RUSLE cover-management factor (C) and compare it with a literature-based benchmark for olive and vineyard systems; and (iii) evaluate Sentinel-2 NDVI-derived vegetation indices as an independent, landscape-scale proxy of ground cover, to support management recommendations for erosion mitigation.

## Methods

### Study area

The study site was a drip-irrigated, six-year-old, SHD olive orchard in a typical, hilly landscape in southwestern Sicily, Italy (37°44’14”N – 12°57’46”E, 220 m a.s.l.). Field activities were conducted on private agricultural land with permission granted by the landowner. No specific permits were required from local or national authorities for the non-invasive soil sampling and measurements, as the work did not involve protected areas, endangered species, or regulated materials.

The climate of the study area, classified as Csa under the Köppen climate classification, is semiarid Mediterranean with a dry period of about 5 months (Mean Annual Temperature: 17.4 ^°^C; Mean Annual Precipitation: 648 mm).

**Fig 1:**
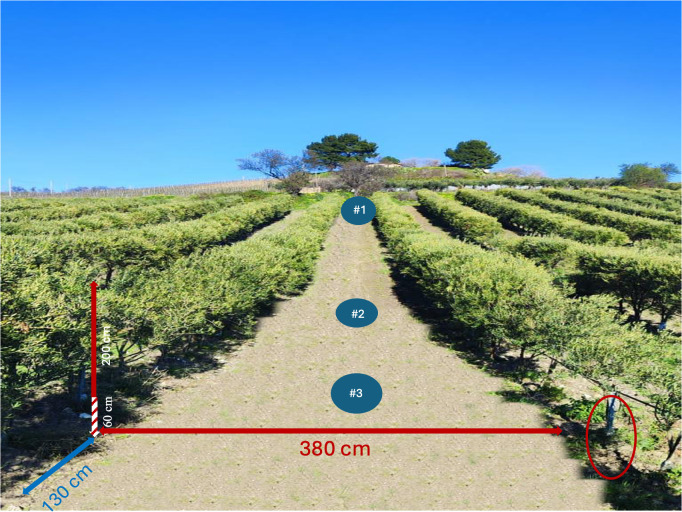
Structural parameters of the studied hedgerow (SHD) olive orchard: olives (spaced 3.8 x 1.3 m and oriented NE-SW) have, on average, tree height = 260 cm, canopy depth = 200 cm, canopy width = 146 cm. The height above soil level maintained free of foliage is 60 cm. Number in the circles indicates the position of soil profiles.

Olives (cv. Lecciana) were planted in 2018, after 80 cm deep ploughing of a 160 m slope topography on a slope of 15.6%, at a plant density of about 2,024 plants ha^-1^ (3.8 x 1.3 m) with NE-SW row orientation along the slope (Fig 1). “Lecciana” is a new low-vigour olive cultivar suitable for super high density orchards obtained from a controlled cross between cv. Arbosana (♀) and cv. Leccino (♂) [3]. Since plantation, the olive orchard has been regularly managed as follows: a total amount of irrigation water of 2,500 m^3^ ha^-1^ year^1^ was supplied in the April-August period; fertilizers application consisted of 0.7 t ha^-1^ as organic fertilization (commercial composted soil improver). The soil has been yearly harrowed 4–5 times with a shallow tillage (10 cm depth) to preserve water evaporation and to control weeds. Pruning and harvest were mechanically performed. A total olive yield of 12 t ha^-1^ was achieved in the last harvest (2023).

At the olive orchard plantation time (t_0_), an espalier structure was built using poles, 120 cm in height, to support the irrigation system. Poles were carefully planted using a pile driver machine to a standard depth of 50 cm and an intra-row distance of 10 m (Fig 1).

Yearly, during the training period, tree pruning was performed to obtain a regular hedgerow shape using a mechanical topping. In 2024, in order to characterize the main vegetative and productive characteristics of the studied SHD olive orchard, including the soil coverage potential of the canopies, a series of plants’ parameters such as trunk cross sectional area, height, width, volume, top canopy surface area, and yield efficiency were determined. The methodologies adopted for measuring such parameters, and related calculations are described in detail in SI.

### Soils description, sampling and analysis

The soils were characterized by the World Reference Base for Soil Resources (WRB) guidelines [20], leading to their classification based on this system. In 2024 three different soil pedons along the slope (Fig 1) were excavated and described. Each pedon was also sampled three times (replications) every 10 cm depth using a core sampler (Ø 8 cm) for soil erosion estimate by isotopic signature method (δ^13^C). Moreover, in a flat olive orchard close to the trial, where neither deposition nor detachment was evident, 10 random samples of topsoil were collected for δ^13^C benchmark determination. Soil samples were air-dried and passed through a 2 mm sieve for laboratory analysis. An EA-IRMS (Elemental Analyser Isotope Ratio Mass Spectrometry, Carlo Erba Na 1500, model Isoprime 2006, Manchester, UK) was used for isotopic analysis. The reference material was IA-R001 (Iso-Analytical Limited wheat flour standard, δ^13^C(V-PDB) = 26.43 m). IA-R001 is traceable to IAEAeCHe6 (cane sugar, δ^13^C(V-PDB) = 10.43 m). IA-R001, IA-R005 (Iso-Analytical Limited beet sugar standard, δ^13^C(VPDB) = 26.03 m), and IA-R006 (Iso-Analytical Limited cane sugar standard, δ^13^C(V-PDB) = 11.64 m) were used as quality control samples. The International Atomic Energy Agency (IAEA), Vienna, distributes IAEA-CH-6 as a reference standard material. The results of the isotope analysis are expressed as δ values relative to the international Pee Dee Belemnite standard as follows:


$$\delta^{\text{1}\text{3}}C(\text{‰})=\frac{R_{s}-R_{st}}{R_{st}}*\text{1}\text{0}\text{0}\text{0}$$ (1)

where R = ^13^C/^12^C, s = sample, and st = standard. Inorganic carbon was removed before stable isotope analysis by acid fumigation following the method of Harris et al. [21].

### Soil erosion quantification by pole height method

To determine erosion rates in the SHD olive orchard, poles were used as erosion markers, according to a procedure adopted for vines and described elsewhere [22]. In 2024, 6 years after the orchard establishment, overground height of 48 poles on three rows was measured checking pole verticality before measuring (Fig 1). For each pole, the difference between the original height (h_t0_) and over-ground height (h_t6_) was used as a marker of the topographical change since the time of olive plantation. Regression variance between pole height and the distance from the top of the profile was performed using the SPSS statistical software version 22 [23].

### Soil erosion quantification by isotopic signature

Isotopic signature (δ^13^C), in the absence of erosion (flat areas), is assumed to be uniformly distributed in the surface layer and its values characterize previous land use and biomass input and remain constant from the olive plantation to trial date, while δ^13^C values are expected to decrease linearly with the soil depth. The used model was developed by Novara et al. [21] to estimate soil redistribution (from the area of detachment to the area of deposition) based on measurements of the variation of δ^13^C between the soil surface and the reference value in the flat area. Organic matter fractionation due to mineralization during an erosion event is not considered determinant because of the limited length of the slope. To calculate the depth of the eroded layer in the top (A_t0_-A_t6_), middle (B_t0_-B_t6_), and bottom (C_t0_-C_t6_) pedons along the profile, δ^13^C enrichment rate was linearly regressed against soil depth:


$$Soildepth(m)=a+b\delta^{\text{1}\text{3}}C$$ (2)

Using this equation at the original value of δ^13^C equal to −27.4 (corresponding to a sampling carried out in a flat olive orchard close to the trial), we obtained the linear original topography of the profile in which the depth of the eroded layer in the top (A_t0_-A_t6_), middle (B_t0_-B_t6_), and bottom (C_t0_-C_t6_) pedons were estimated as the intersection among these three linear regressions (eq. 3) and the δ^13^C original values (−27.4) (Fig 4):


$$Soillosses(m)=a+b\delta^{\text{1}\text{3}}Coriginalvalue$$ (3)

The soil detachment and deposition areas, represented by irregular polygons, were calculated according to the Gauss formula since their vertices were known (Fig 5).

Assuming that a polygon has vertices (x1,y1), (x2,y2),…, (xn,yn) and that (xn + 1,yn + 1)=(x1,y1), the area of the polygons is given by:


$$Detachmentarea\left(m^{\text{2}}\right)=\frac{\sum_{\text{5}}^{\text{1}}\left(xiyi+\text{1}-xi+\text{1}yi\right)}{\text{2}}$$ (4)

$$Depositionarea\left(m^{\text{2}}\right)=\frac{\sum_{\text{3}}^{\text{1}}\left(xiyi+\text{1}-xi+\text{1}yi\right)}{\text{2}}$$ (5)

The volume of net soil erosion (SE) in Mg ha^-1^ was calculated as follows:


$$SE\left(Mg{ha}^{-\text{1}}\right)=\frac{\left(Detachmentarea\left(m^{\text{2}}\right)-Depositionarea(m^{\text{2}})\right)*\text{1}\text{0}\text{0}\text{0}\text{0}\left(m^{\text{2}}\right)*BD(gcm^{\text{3}})}{Interrowwide(m)}$$ (6)

where soil bulk density (BD) was measured at 10 cm depth in three sampling points for each pedon using the core method [24] to transform the calculated volume into weight (Mg ha^-1^).

### *Cover management (*C_factor_)

The RUSLE scheme was applied to estimate the C_factor_:


$$C_{factor}=\frac{SE}{KRLSP}$$ (7)

where SE is soil loss (Mg ha^-1^ year^-1^), R is the rainfall factor (MJ mm ha^-1^ h^-1^ year^-1^), K is the soil erodibility factor (Mg ha h MJ^−1^ h^−1^ mm^−1^), LS is the topographic factor, and P is the support practice factor. The C_factor_, which represents the influence of vegetation on soil erosion, ranges from 0 to 1.

To estimate the C_factor_ in the RUSLE model, several subfactors need to be known, such as past management practices, vegetation height, surface cover, and roughness (details in SI, S2).

A list of values for C_factor_ for traditional olive orchards and vineyards was obtained by reviewing selected, updated literature, including available data from activities carried out in the same area of this experiment (Table 1).

**Table 1:** Reported C_factor_ values for olive orchards and vineyards, according to different Authors, and average C_factor_ values.

Crop	C_factor_ value	Reference	Average value
Olive orchard	0.1-0.3	[25]	0.15
	0.1	[16]	
	0.16; 0.25; 0.41^*^	[26]	
	0.005; 0.007; 0.02; 0.1; 0.11; 0.21; 0.29	[27]	
Vineyard	0.34	[25]	0.3
	0.35	[28]	
	0.18; 0.3; 0.4	[29]	
	0.3	[30]	
	0.1; 0.12; 0.18; 0.22; 0.23	[17]	

*SHD olive orchard.

### *C*_*factor*_ *determination from NDVI*

Many authors have suggested evaluating the C_factor_ through the Normalized Difference Vegetation Index (NDVI) obtained from satellite imagery [31–33]. This index is calculated from multispectral sensors to capture reflectance images in red (RED) and infrared (NIR) spectral regions:


$$NDVI=(\frac{NIR-RED}{NIR+RED})$$ (8)

Sentinel-2 NDVI time-series (average data from September to March), with spatial resolution equal to 10 m, were utilized. To transform the NDVI values in C_factor_, the following equation [33] was applied:


$$C_{factor}=exp-\text{2}\left(\frac{NDVI}{\text{1}-NDVI}\right)$$ (9)

### Methodological framework

**Fig 2:**
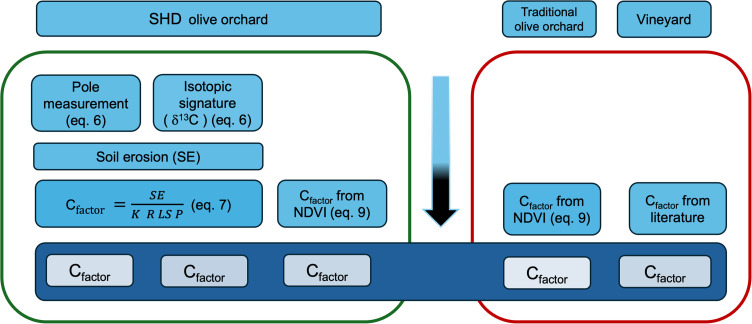
Schematic representation of methodological approaches followed for C_factor_ determination. The two boxes are intended for comparison among crop systems: the left box represents the 3 methods adopted for C_factor_ calculation in tested SHD olive orchard; the right box reports the C_factor_ calculation for traditional olive orchard and vineyard. The vertical blue arrow indicates the sequence of the methodological framework adopted.

The use of C_factor_ to compare the performances of different orchard systems lies in the fact that standardization allows easy comparison of erosion capacity control (Fig 2).

C_factor_ was calculated for SHD and traditional olive orchards using the RUSLE equation and the parameters previously used in the same environment [22] according to eq. 7, and from Sentinel-2 images, according to eq. 9. The three C_factors_ values were also compared together with C_factors_ determined for traditional olive orchard and vineyard systems using NDVI and data from literature review (Fig 2).

## Results

### SHD structure: canopy architecture and tree production performance

The main vegetative and productive characteristics of the studied SHD olive orchard are reported in Table 2.

**Table 2:** Main vegetative-productive characteristics of the SHD olive orchard studied with a density of 2,024 trees ha^-1^. The data refers to the 6^th^ year after planting (2024). Vegetative values are means of 15 trees. Productive values are related to the last harvest (total yield = 12 t ha^-1^). s.d. = standard deviation.

	Tree height(cm)	Canopy depth (cm)	Canopy width (cm)	Canopy volume (m^3^)	Canopy volume (m^3^ ha^-1^)	Covered ground per tree (m^2^)	TCSA* per tree (dm^2^)	Yield per tree (g)	Yield efficiency (g dm^-2^)
	260.0	200.3	146.0	3.8	7,697.1	1.90	0.63	5,930	9,442.7
s.d.	10.0	14.9	8.3	0.4	717.6	0.1	0.17	n.a.	n.a.

*TCSA: trunk cross sectional area; n.a.: not available.

The vegetative measured parameters are in accordance with the architectural model generally required (in terms of tree height and width) suitable for intensive hedgerow olive plantings to be harvested with straddle machines [12]. As far as the estimate of the soil coverage offered by the canopies is concerned, calculations performed using the above reported measurements allowed us to estimate a ground canopy coverage of approximately 38%, and, therefore, a percentage of uncovered bare soil of approximately 62%. The production parameters are in line with the expectations for SHD systems under similar conditions [34] and with the results obtained by other authors in similar environments [35–36].

### Soil pedon

The three soil pedons excavated along the profile from its top to the toe, positioned as detailed below, showed distinct properties (S1 Table, S1 Fig). The soils at the top position (#1) denote characteristics of youthful soil with fine-textured particles resulting from the gradual degradation of parent material by water and erosion (Cambisols). The landscape’s structure is shaped by drainage conditions, which in turn influence weathering intensity, elevation changes, and the redistribution of weathering products along the slope. Erosion transports finer materials downhill, altering the taxonomy of the slope. Pedon, at the base of the slope (#3) starts to meet the criteria for Vertisols. Here, fine materials undergo weathering and aeration, resulting in greater water accumulation compared to the top pedon, even during dry periods.

### Soil erosion quantification by the pole height method

**Fig 3:**
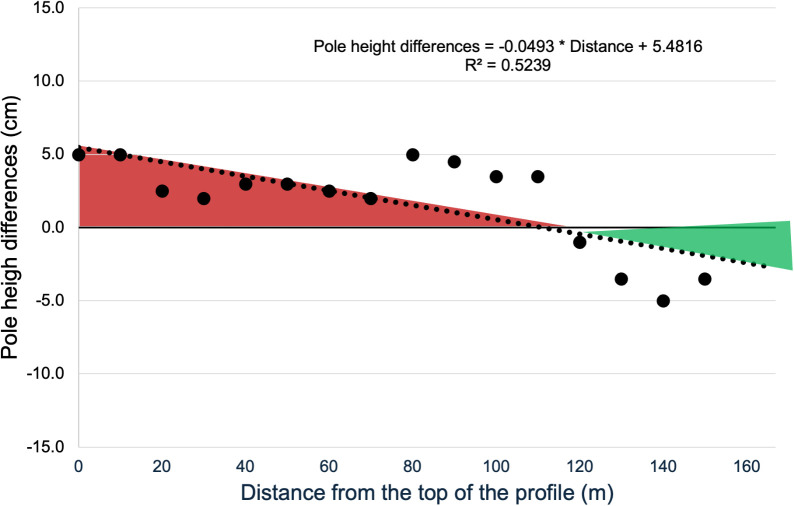
Pole height variation observed in 2024, six years after the pole installation (black dots) and pole height regression against the distance from the top of the profile (black dotted line). Negative values indicated areas of deposition (green), while positive values indicated areas of detachment (red).

The applied pole height method allowed the appreciation of the variation of soil profile morphology, (deposition and detachment areas), following erosion processes occurred in the study site since the year of SHD plantation. The variation in pole height between the year of observations (2024) and the pole installation (2018) showed the change occurred in the profile topography in the considered time period (6 years) (Fig 3). The pole height difference ranged from −5 cm to +5 cm, as shown in Fig 3.

The linear regression between pole height and its distance from the top of the slope profile (Fig 3, top) showed a high statistical significance (Table 3) and permitted to clearly discriminate detachment areas from deposition ones also thanks to the absence of irregularities along the profile (no convexities, nor concavities).

**Table 3:** Analysis of variance (ANOVA) of the regression between pole height and distance from the top of the profile.

	df	MS	F	p
Regression	1	14970.7	14.9	0.002
Residual	13	1002.2		
Total	14			

The total amount of soil erosion (Mg ha^−1^) at the end of the 6-year period was calculated as the difference between the erosion and the deposition for the whole plot (eq. 6). On average, the annual erosion rate was 28.8 Mg ha^−1^ yr^−1^ as obtained from the difference between 41.9 Mg ha^−1^ yr^−1^ of detached soil and 13.1 Mg ha^−1^ yr^−1^ of deposed soil.

### Soil erosion quantification by isotopic signature

**Fig 4:**
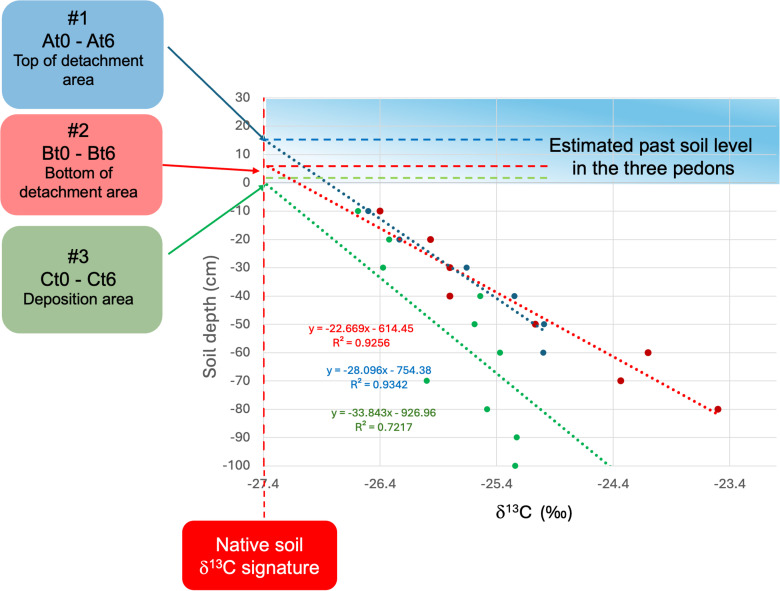
Regressions ofδ^13^C and soil depth in the three pedons. The height of the past soil level can be read in the intercept of each regression on the vertical red dashed line indicating the native (flat area with no erosion processes used as a benchmark) soil δ^13^C signature equal to −27.4‰ (see eq. 3).

The changes of δ^13^C values in the soil profiles and along the slope indicate that erosion and accumulation have occurred since the time of plantation in 2018. The δ^13^C values are lower in the topsoil in all pedons than in the topsoil of the flat area close to the plot trial used as a benchmark (δ^13^C = −27.4), indicating that the profile was eroded (Fig 4).

The three regressions between δ^13^C values and soil depth were all characterized by significant R^2^ values (Fig 4).

**Fig 5:**
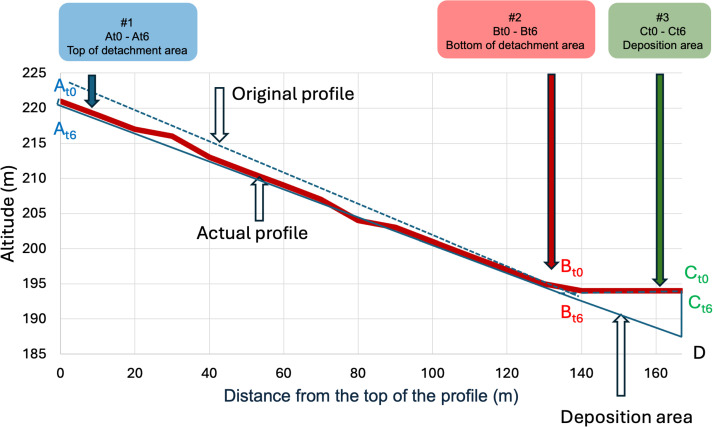
Representation of theδ^13^C approach to reconstruct the original profile. #1, #2 and #3 represent pedons’ positions. Xt0-Xt6 are the thicknesses of the soil eroded calculated by isotopic signature along the 6-year period (2018-2024).

According to the proposed methodology, the intercept was calculated using eq. 3 forcing the δ^13^C value to non-eroded (native soil δ^13^C signature). The results showed profile differences between original and actual profiles of −0.15, −0.08, and +0.05 m at the top and bottom of the detachment area and at the deposition area, respectively. These results allowed the reconstruction of the original profile of the slope showed in Fig 5. The total amount of soil erosion (Mg ha^−1^) at the end of the 6-year period was calculated (eq. 6). On average, the annual erosion rate was 30 Mg ha^-1^yr^-1^, as obtained from the difference between a soil detachment of 49 Mg ha^-1^yr^-1^ and a soil deposition of 19 Mg ha^-1^yr^-1^.

### *C*_factors_ *determination*

#### *Bibliographic* C_factors_ *comparison.*

Table 1 showed how olive orchards and vineyards can differently influence C_factor_. On average, the two different datasets showed values of 0.15 ± 0.1 and 0.3 ± 0.08 for olive and vineyard, respectively. The reported values for vineyards range widely from as low as 0.1 to as high as 0.4, while the values for olive orchards range from very low (0.005) to relatively high (0.41), indicating strong differences exerted by different orchard systems. In particular, it is worthwhile noting that the highest C_factor_ (0.41) reported by Rodríguez Sousa et al. [26] is the only one referred to SHD olive orchards, implying higher potential soil loss of this last system than traditional ones and similar to that of vineyards.

### Comparative analysis of soil erosion estimation methods

**Fig 6:**
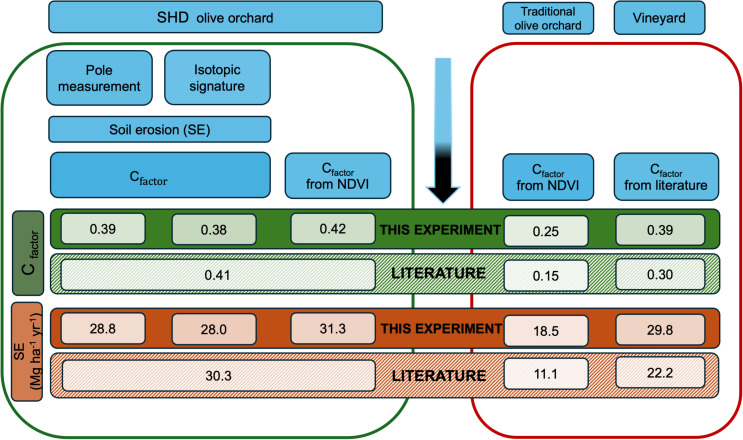
Comparative analysis of soil erosion estimation methods: C_factors_ in SHD olive orchard (present study), traditional olive orchard and vineyard from selected bibliographic sources (Table 1) and NDVI according to the methodological approach proposed and soil erosion quantification (SE).

From soil erosion estimated by the pole method and isotopic signature according to eq. 7 the corresponding values of C_factors_ were calculated, yielding values of 0.39 and 0.38 for pole and isotopic method, respectively (Fig 6). Also C_factor_ estimated by NDVI from Sentinel 2 data revealed similar values when compared to those obtained from both pole and isotopic methods and comparable to the corresponding available bibliographic data. Further, also values of intensive olive orchard (SHD) and vineyard resulted comparable.

## Discussion

Consistent results were obtained across the pole height and isotopic (δ^13^C) approaches, indicating unsustainable soil erosion rates in the studied SHD orchard (~29–30 Mg ha ⁻ ¹ yr ⁻ ¹), which are 154% higher than values typically reported for conventional (non-SHD) olive orchards [16]. This convergence increases confidence in the estimates and supports the interpretation that SHD orchards established on semi-arid slopes can experience soil losses comparable to vineyards in the same environment [22].

A key driver of this high erosion susceptibility is the downslope orientation of rows required for straddle harvesting. This orientation constrains machinery traffic and tillage to be conducted downslope, thereby enhancing tillage erosion, concentrating overland flow, and promoting rill development, as previously observed in steep-slope vineyards [37–39]. In addition, SHD hedgerows provide limited soil protection because narrow, low canopies are maintained for mechanized harvesting. In our site, canopy ground cover was ~38%, leaving ~62% bare soil. Such limited cover—together with relatively porous crowns typical of hedgerow training [15,35]—reduces rainfall interception and increases detachment and transport during erosive events.

Frequent downslope tillage for weed control and evaporation reduction further increases erosion susceptibility, highlighting the need to reduce excessive tillage and adopt conservation practices on slopes >10% [6,40,41]. Practical options include permanent or seasonal cover crops, retention/incorporation of pruning residues (mulching), and vegetated barriers aligned along contour and/or placed across the slope, which have proven effective in Mediterranean orchards and vineyards [5,42–44] and are increasingly considered feasible in SHD systems [45–46].

### Limitations

This study was conducted in a single SHD orchard and over a specific topographic and management setting (semi-arid climate, ~ 15.6% slope, downslope operations). Therefore, the magnitude of erosion and the derived C_factor_ should be interpreted as site-specific and not necessarily representative of all SHD orchards. Moreover, the absence of a paired, co-located traditional/intensive olive control limits direct attribution of differences to planting system alone. Finally, Sentinel-2 NDVI (10 m) may partially mix canopy and inter-row signals in narrow hedgerows; however, this is consistent with the RUSLE C_factor_, which represents the whole orchard system (row plus inter-row). We therefore used NDVI as an independent proxy alongside field-based estimates, and future studies should validate cover metrics with higher-resolution remote sensing (e.g., UAV) and multi-year monitoring across contrasting soils, slopes, and management practices.

## Conclusions

This study represents a first attempt in Italy to quantify soil erosion in super-high-density (SHD) olive orchards by combining isotopic (δ^13^C) soil signatures, erosion-pole measurements, and satellite-derived vegetation indices. In the studied semi-arid sloping site, erosion rates were comparable to vineyards and markedly higher than those reported for traditional olive systems. Row orientation conducted downslope, together with reduced canopy protection, substantially increases erosion susceptibility in SHD orchards on sloping terrain. Given the rapid expansion of SHD orchards, their environmental impacts—particularly on fragile Mediterranean slopes—remain insufficiently assessed. While intensification can increase oil production, this trend may not extend to oil quality or typicity, and may accelerate the loss of traditional systems and associated genetic resources and landscapes. Caution is warranted when converting traditional olive orchards to SHD systems on marginal terrain. To reduce soil losses, conservation practices such as mulching/pruning-residue retention, cover cropping, and contour-aligned ground operations should be considered. However, further investigation is needed to optimize these practices under specific pedoclimatic conditions and to assess long-term impacts on yield quantity and quality.

## Supporting information

S1 Plants Plant measurements and calculations (e.g., TCSA, canopy volume, top canopy area) used to estimate ground cover. (DOCX)

S1 RUSLE Details of RUSLE factor estimation (R, K, LS, P) and subfactors used for the cover-management factor (C). (DOCX)

S1 Soils WRB-based field description of the three pedons excavated along the slope; and soil classification of the three pedons according to IUSS Working Group WRB (2022). (DOCX)

S1 Table WRB-based field description of the three pedons excavated along the slope. (DOCX)

S1 Fig Soil classification of the three pedons according to [20]. (DOCX)

## Abbreviations

DEM: Digital elevation model: raster representation of ground-surface elevation
NDVI: Normalized difference vegetation index (NIR − Red)/(NIR + Red) proxy for vegetation greenness and vigor
RUSLE: Revised Universal Soil Loss Equation: empirical model estimating long-term average annual soil loss by water erosion
SHD: Super high-density (planting): very high-density hedgerow planting system (commonly used in orchards/olive groves)
TCSA: Trunk cross-sectional area: trunk basal area measured at a specified height; proxy for tree size/vigor
UAV: Unmanned Aerial Vehicle
WRB: World Reference Base for Soil Resources: international soil classification and correlation system (IUSS)
