# Supplementary material for: Olive orchard intensification compromises soil water erosion control in a semi-arid environment
Source: PLoS One. 2026 Apr 30;21(4):e0346675. doi: 10.1371/journal.pone.0346675 (PMC13132176; doi:10.1371/journal.pone.0346675)
Supplement: S1 Plants — (DOCX) [file pone.0346675.s001.docx]

**Supplementary Information**

**S1 Plants. Plant measurements and calculations used to estimate ground cover**

*Trunk circumference was measured 0.35 m above the ground for the calculation of trunk sectional area (TCSA). The average canopy volume and the top canopy surface area (used for estimating ground coverage) were calculated from measurements of height and width of the canopy assuming a regular rectangular canopy shape. The yield per tree was obtained by dividing the total yield per hectare (12 t ha^-1^) by the number of plants per hectare (2024). Yield efficiency per tree was calculated as fruit yield (g) / TCSA (dm^2^). All measurements regarding vegetative parameters were taken in May 2024 on three groups of five trees each located in the top, middle and bottom of a single central row.*
