# Supplementary material for: Olive orchard intensification compromises soil water erosion control in a semi-arid environment
Source: PLoS One. 2026 Apr 30;21(4):e0346675. doi: 10.1371/journal.pone.0346675 (PMC13132176; doi:10.1371/journal.pone.0346675)
Supplement: S1 RUSLE — (DOCX) [file pone.0346675.s002.docx]

**Supplementary Information**

**S2 RUSLE. Details of RUSLE factor estimation (R, K, LS, and P) and subfactors used for the cover-management factor (C)**

*Rainfall erosivity (R) Rainfall erosivity is the kinetic energy of a raindrop’s impact and the rate of associated runoff. Among the factors used within RUSLE and its earlier version, the Universal Soil Loss Equation (USLE), rainfall erosivity is of high importance as precipitation is the driving force of erosion and has a direct impact on the detachment of soil particles, the breakdown of aggregates and the transport of eroded particles via runoff. The R-factor accumulates the rainfall erosivity of individual rainstorm events and averages this value over multiple years. Rainfall erosivity data was derived from Panagos et al. (2015).*

*Soil erodibility (K) Application of the RUSLE model also required the K factor estimation, which represents the influence of different soil properties on slope susceptibility to erosion (Renard et al., 1997). K factor also defines the “mean annual soil loss per unit of rainfall erosivity for a standard condition of bare soil, recently tilled up down slope with no conservation practice” (Morgan, 2005).*

*Slope length and steepness factor (LS) A high-resolution digital elevation model (DEM) was used to assess the LS factor of each plot. LS factor was calculated according to Renard et al. (1997).*

*Conservation practice (P) Conservation practice (P) was set at a value equal to unity (Adornado et al., 2009). The absence of any conservation practices in the specific area indicated that a value of 1 would have no significant impact on the Wischmeier equation, as suggested by Panagos et al. (2015).*
