# Supplementary material for: Olive orchard intensification compromises soil water erosion control in a semi-arid environment
Source: PLoS One. 2026 Apr 30;21(4):e0346675. doi: 10.1371/journal.pone.0346675 (PMC13132176; doi:10.1371/journal.pone.0346675)
Supplement: S1 Soils — (DOCX) [file pone.0346675.s003.docx]

**Supplementary Information**

**S3 Soils. WRB-based field description of the three pedons excavated along the slope; and soil classification of the three pedons according to IUSS Working Group WRB (2022).**

*Pedon #1 and #2, Cambisols display characteristics of a youthful soil with few signs of distinct soil layers evident, and no clear demarcations between the various hues. The soil profile, representing a typical soil undergoing transformation, exhibits a fine-textured particles, resulting from gradual degradation of parent material by water and erosion. The landscape's structure within this catena is influenced by eluvial and illuvial processes, which, in turn, are affected by drainage conditions. These conditions are determined by surface and subsurface water movement, matter flux, and energy exchange at ground level. All these factors impact weathering intensity, elevation changes, and the redistribution of weathering products along the profile. The finer materials, in this case inherited from the parent material, following transport during the erosive phenomenon, accumulate at the base of the profile, even transforming its taxonomy. Pedon #3, at the base of the profile, begins to meet the requirements to be a Vertisol. Here the fine materials, during pedogenesis, aerate from both below and above, and in situ undergo weathering which transforms the phyllosilicates towards more expandable 2:1 types. The accumulation of water, even in the driest periods, in these soils is greater than in the summit Cambisols.*
