# Supplementary material for: Olive orchard intensification compromises soil water erosion control in a semi-arid environment
Source: PLoS One. 2026 Apr 30;21(4):e0346675. doi: 10.1371/journal.pone.0346675 (PMC13132176; doi:10.1371/journal.pone.0346675)
Supplement: S1 Table — (DOCX) [file pone.0346675.s004.docx]

**Supplementary Information**

**S3 Soils.**

**Table S1.** WRB-based field description of the three pedons excavated along the slope.

*Descriptive data of the three pedons excavated in the study site according to WRB (IUSS WG WRB, 2022). Landform&topography: gradient upslope 19%, downslope 10%, aspect 45°, shape VV. Climate&weather: Csa, ecozone SWR; weather condition PC, past weather condition RH. Vegetation&landuse: strata MS, type CPP, IR. Surface: CSF/C/S; surface cracks N (reversible); water above N; uneveness N. Erosion: WS, S, PR. Parent material: Piacenzian-Gelasian.*

| Pedon | m a.s.l. | horizon | ^1^ul cm | ^1^ll cm | ^2^bound | ^3^frag | ^4^text | ^5^struct | ^6^pores | ^7^cracks |  | ^8^colour |  |
| --- | --- | --- | --- | --- | --- | --- | --- | --- | --- | --- | --- | --- | --- |
| #1 | 218 | Ap | 0 | 20 | a, s |  | L | CL, W, ME | DT, FI/ME, C |  |  | 7.5YR5/4 |  |
|  |  | Bw | 20 | 40 | c, s |  | CL | BS, M, ME | DT, FI/ME, F |  |  | 7.5YR3/4 |  |
|  |  | Bi | 40 | 70 | v, s | FA, F | C | PR, M, ME | DT, FI/ME, VF | RT, HC, ME, F |  | 7.5YR3/2 |  |
|  |  | Ck | 70 | + |  | CA, F | C | MR, S |  |  |  | 2.5Y8/6 |  |
| #2 | 203 | Ap | 0 | 20 | a, s |  | L | CL, W, ME | DT, FI/ME, C |  |  | 7.5YR5/4 |  |
|  |  | Bw | 20 | 50 | c, s |  | CL | BS, M, ME | DT, FI/ME, F |  |  | 7.5YR3/4 |  |
|  |  | Bi | 50 | 75 | v, s | FA, F | C | PR, S, ME | DT, FI/ME, VF | RT, HC, ME, F |  | 7.5YR3/2 |  |
|  |  | Cmg | 75 | + |  |  | C | MR, S |  |  |  | 2.5Y8/6 |  |
| #3 | 200 | Ap | 0 | 30 | a, s |  | CL | CL, M, ME | DT, FI/ME, F |  |  | 7.5YR5/4 |  |
|  |  | Bw | 30 | 50 | c, s | FA, F | C | BS, M, ME | DT, FI, VF | RT, HC, ME, F |  | 7.5YR3/4 |  |
|  |  | Bi1 | 50 | 80 | c, s | FA, F | C | PR, S, ME | DT, FI, VF | RT, HC, ME, C |  | 7.5YR3/2 |  |
|  |  | Bi2 | 80 | + |  |  | C | PR, S, ME |  |  |  | 7.5YR3/2 |  |

^1^ upper limit, lower limit; ^2^ boundary; ^3^ coarse fragments, weathering stage; ^4^ soil texture; ^5^ structure; ^6^ non-matrix pores; ^7^ cracks persistence and continuity; ^8^ Munsell soil colour (moist).
