# Supplementary material for: Olive orchard intensification compromises soil water erosion control in a semi-arid environment
Source: PLoS One. 2026 Apr 30;21(4):e0346675. doi: 10.1371/journal.pone.0346675 (PMC13132176; doi:10.1371/journal.pone.0346675)
Supplement: S1 Fig — (DOCX) [file pone.0346675.s005.docx]

**Supplementary Information**

**S3 Soils.**

**Figure S1.** Soil classification of the three pedons according to IUSS WG WRB (2022).

| #1 | #2 | #3 |  |
| --- | --- | --- | --- |
| Eutric Cambisol (Clayic) | Vertic Cambisol (Clayic) | Haplic Vertisol (Gleyic) |  |
| 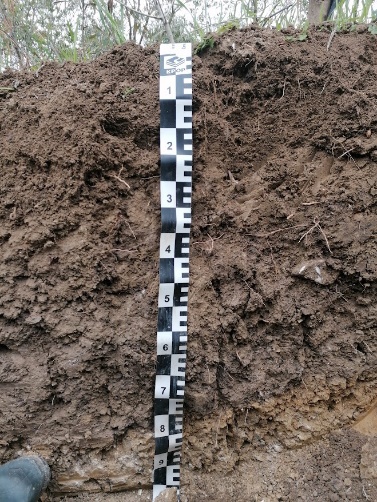 | 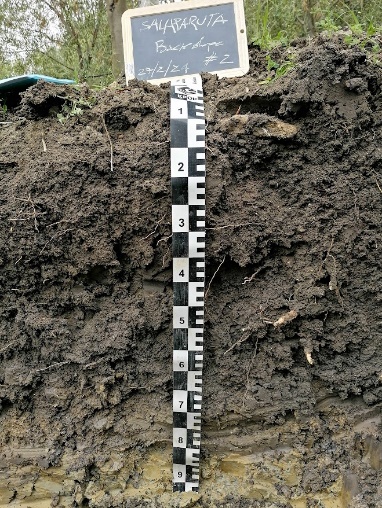 | 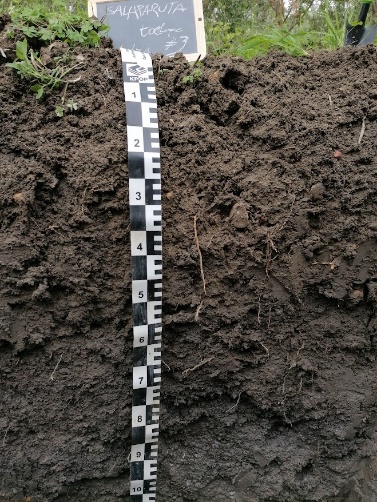 | |
